# Order of the major constituents in sign languages: implications for all language

**DOI:** 10.3389/fpsyg.2014.00376

**Published:** 2014-05-12

**Authors:** Donna Jo Napoli, Rachel Sutton-Spence

**Affiliations:** ^1^Department of Linguistics, Swarthmore CollegeSwarthmore, PA, USA; ^2^Post-Graduate Department of Translation, Federal University of Santa CatarinaFlorianopolis, Brazil

**Keywords:** sign languages, word order, vision, syntax, sensorimotor systems and language, gesture

## Abstract

A survey of reports of sign order from 42 sign languages leads to a handful of generalizations. Two accounts emerge, one amodal and the other modal. We argue that universal pressures are at work with respect to some generalizations, but that pressure from the visual modality is at work with respect to others. Together, these pressures conspire to make all sign languages order their major constituents SOV or SVO. This study leads us to the conclusion that the order of S with regard to verb phrase (VP) may be driven by sensorimotor system concerns that feed universal grammar.

## Introduction

In the initial period of linguistic analysis of sign languages, scholars tended to stay away from examining phenomena that were modality bound in favor of those that were more universal, in order to establish that sign languages were bona fide languages (see Woll, 2003 for an overview). Since the mid-1980s, however, scholars have turned their attention to the importance of modality (Bergman and Wallin, 1985; Sze, 2003; also see Meier et al., 2002).

We focus attention on the issue of sentence-level sign order in sign languages, looking at subject, object and verb. Research on 42 sign languages (see Table 1), taken as a whole, coupled with our own observations leads to generalizations about order that contrast to varying degrees with word order in spoken languages. We consider two hypotheses: (1) that our generalizations are due to universal pressures on language, ones which are seen most strongly in young languages, and (2) that our generalizations are due to modality; that is, the patterns for sign order in sign languages are determined by what makes sense visually. We conclude that the first hypothesis carries us quite far, but consideration of visual pressures allows us to account for all the observed tendencies in our study. We conclude that all sign languages should order their constituents SOV and SVO in most declaratives. Importantly, this does not preclude the possibility that languages may impose language-specific constraints on order within a phrase (see work on noun phrases in Estonian Sign Language, Miljan, 2000, and Taiwanese Sign Language, Zhang, 2007).

**Table 1:** **Sign Languages**.

Adamorobe	Nyst, 2007
Al-Sayyid Bedouin	Sandler et al., 2005; Padden et al., 2010
American	Fischer, 1975, 1990; Friedman, 1976; Klima and Bellugi, 1979; Baker and Cokely, 1980; McIntire, 1980; Woodward, 1980; Liddell and Johnson, 1986; Lillo-Martin, 1986, 1991; Wilbur, 1987, 2002, 2003; Padden, 1988, 1990; Fischer and Janis, 1990; Kegl, 1990; Liddell, 1990; Petronio, 1993; Bahan, 1996; Matsuoka, 1997; Neidle et al., 2000; Taub, 2001; Aronoff et al., 2003; Chen Pichler et al., 2008
Argentine	Massone and Curiel, 2004
Australian	Johnston and Schembri, 2007a,b; Johnston et al., 2007
Austrian	Wilbur, 2002; Chen Pichler et al., 2008
Brazilian	Quadros, 2003; Quadros and Quer, 2008
British	Deuchar, 1983; Sutton-Spence and Woll, 1999
Catalan	Morales-López et al., 2005; Quadros and Quer, 2008
Chinese	Yau, 2008
Colombian	Oviedo, 2001
Croatian	Milkovic et al., 2006, 2007; Chen Pichler et al., 2008
Danish	Engberg-Pedersen, 1994; Kristoffersen, 2003
Estonian	Miljan, 2000
Finnish	Jantunen, 2008
Flemish	Vermeerbergen, 1996; Johnston et al., 2007; Vermeerbergen et al., 2007a,b
French	Baron, 1998; Cuxac, 2000; De Langhe et al., 2004; Cuxac and Sallandre, 2007
French Swiss	Boyes Braem et al., 1990
German	Glück and Pfau, 1998; Leuninger, 2000; Hänel, 2005; Happ and Verköper, 2006; Plaza-Pust, 2008; Plaza-Pust and Weinmeister, 2008
Greek	Efthimiou and Fotinea, 2007
Hong Kong	Sze, 2003
Indian	Zeshan, 2003; Aboh et al., 2005
Inuit	Schuit et al., 2011
Irish	Johnston et al., 2007
Israeli	Meir, 1995; Aronoff et al., 2003; Rosenstein, 2004; Meir and Sandler, 2007; Meir et al., 2007, 2010a,b
Italian	Volterra et al., 1984; Corrazza et al., 1985; Boyes Braem et al., 1990
Japanese	Nakanashi, 1994
Kenyan	Akach, 1992; Jefwa, 2009
Malagasy	Minoura, 2008
Mexican	Quinto, 1999
New Zealand	McKee and Kennedy, 2005
Polish	Farris, 1994; Wojda, 2010
Portuguese	Delgado-Martins et al., 1994
Providence Island Language	Washabaugh et al., 1978; Woodward, 1987
Quebec	Nadeau and Desouvrey, 1994
Russian	Kimmelman, 2012
The Netherlands	Bogaerde and Mills, 1994; Coerts, 1994
South African	Vermeerbergen et al., 2007a,b
Spanish	Bobillo-García et al., 2006; Morales-López et al., 2012
Swedish	Bergman and Wallin, 1985
Taiwanese	Smith, 2005; Zhang, 2007; Tsay and Myers, 2009
Turkish	Zeshan, 2006

## Terminology regarding predicates and nominals

We use V throughout to indicate predicates of any category. We use S and O to refer to the arguments of a V, but these labels are problematic, since what is referred to as S in the literature is typically agent and what is referred to as O is typically any other argument. We do not include discussion of non-argument nominals.

As for nominals, to understand the generalizations here we must pay attention to articulation. Referents can be manually articulated via a lexical NP (including fingerspelling) or via finger pointing to an object within sight. These are two typical ways of introducing referents (what we here call players) into the discourse (what we here call the conversational scene).

Once a player is on the scene, it is commonly assigned a spatial index and subsequently this index is pointed to Johnston (2013). Many behaviors fall under the rubric “pointing”: referential spatial indexes can be pointed to by finger, gaze, lip, chin, head-tilt, among others. Further, already introduced arguments can be incorporated into a V (Wilbur, 2003), or indicated by body shift (Bahan, 1996) and/or embodiment by the signer (Meir et al., 2007). For justification of including all these mechanisms as ways to indicate arguments, see Neidle et al. (2000). Still, null arguments are possible (Lillo-Martin, 1986). Where a sentence appears to have an “omitted” argument (i.e., no articulatory realization, manual or non-manual), we take such an argument to be expressed earlier in the discourse or to be understood through context, otherwise the sentence would be incomprehensible (Bergman and Wallin, 1985, p. 220). Argument omission is typical with a series of verbs that have the same argument (often S), where that argument has already been established (McIntire, 1980; Padden, 1988). Note that “I” and “you” are always on the scene, since they are participants in the sign/speech act.

Since ways of referring to old-information referents are, with one exception, layered (i.e., built into the V or indicated by the non-manuals), one cannot talk about their order with respect to the V: they are expressed simultaneously. We understand these referents in the context of the discourse and of our knowledge of who the signer/speaker is, and what the signer/speaker might be trying to communicate; this is a general practice in language comprehension (Carston, 2002, among others).

The only exception is manual (i.e., finger or hand) pointing; this is generally not simultaneously articulated with the V. Many of our sources do not indicate manual pointing, but we use any information they do present. We categorize lexical NPs and NPs indicated by manual pointing together under the rubric “manually-expressed NPs,” and we use the abbreviation MNP.

## A word on data

We surveyed articles on 42 sign languages, as shown in Table 1, where language names are given in English (cited studies tell which varieties of language are gathered under these rubrics). We draw upon data collected and analyzed in these works as well as cite insights of others, without necessarily adopting the authors' analyses.

While some conclusions in these works seem resilient within the study of a given language and sometimes across languages, many are fragile in that they do not find corroboration in other studies. Brennan (1994) points out that American Sign Language, for example, has been analyzed as SVO (Fischer, 1975), V-final (Friedman, 1976), and topic-comment (Baker and Cokely, 1980). We add that ASL has been analyzed as varying between SVO and SOV depending on sociolinguistic factors (Woodward, 1980). Further, sometimes no constraints on word order emerge; in Malagasy Sign Language all possible permutations of S, O, and V occur (Minoura, 2008). Bouchard and Dubuisson (1995) and Bouchard (1996) argue that there is no base order in sign languages (and they say spoken language has this option, as well), looking at ASL and Quebec Sign Language.

Unfortunately, much of the confusion in the literature results from how the various studies were carried out. While replication of results is a revered principle in science, many times the best we can hope for is corroboration (Giles, 2006). But often not even corroboration is found on sign order. Johnston et al. (2007; see also Coerts, 1994) point out that attempts at comparing studies are confounded by the range of methodologies adopted in data collection, varying from elicitation based on drawings, to translations of sentences in a written language, to seeking grammaticality judgments of constructed sentences, to examining spontaneous or naturalistic data (monologs or dialogs).

Reliance on these methods, rather than on a large corpus of naturally occurring data gathered with no aim other than general linguistic study, is problematic (McEnery and Wilson, 1996). Such methods' reliability is even more doubtful for sign language study, where often the number of native signers consulted is small (Johnston and Schembri, 2007a). The sociolinguistics of Deaf communities complicates the issue further. Sign language communities are small minority communities whose language is young and without well-developed community-based standards of correctness and which have few true native signers (Johnston, 2013). Concerns about basing analyses of any language on very limited data and about what we can conclude from different methods of data collection abound (Sprouse, 2011; Weskott and Fanselow, 2011; Gibson and Fedorenko, 2013) and lead to the conclusion that methodological options in accumulating evidence for syntactic analysis should be expanded.

With regard to sign order studies, Johnston et al. (2007) point out further that often information about the linguistic consultants that might be pertinent to language variation is not given, and that issues as fundamental as having consistent criteria (or even any explicit criteria) for what counts as a clause or a complete sentence remain unresolved (and see Crasborn, 2007; Jantunen, 2008). Here we take the relevant unit for discussion to be predicates and their constellations of arguments, regardless of repetition of various parts (as in V sandwiching/doubling, see Fischer and Janis, 1990; Kegl, 1990; Matsuoka, 1997). We take a light V and the main V it supports to be one predicate, an unproblematic analysis since no arguments intervene between the two in the data observed (as in signing GIVE plus HUG, rather than simply HUG—a rare construction, reported for Flemish Sign Language, but which might reveal spoken language influence, see Johnston et al., 2007).

The variety of theoretical approaches used, from syntactically based ones to semantically-pragmatically based ones, is another complicating factor (Johnston et al., 2007). Theoretical biases impose themselves in fundamental ways. Simply transcribing sign languages with a morpheme-by-morpheme gloss and then a translation into a spoken language can obscure the information (lexical and functional) in a sign and how it is packaged (Slobin, 2006); there is no way to represent linguistic data that is theory-neutral (Ochs, 1979). Thus, in any given study we may not know exactly what data are under consideration and, hence, exactly what we can conclude. Further, many of the findings in the various studies consist of generalizations often in the form of tables that give numbers of occurrences of templates such as OV, SOV, SVO, etc., but few actual examples, so that various comparisons we wanted to make were precluded. Given this lack of information, we have no choice but to transcribe sign streams in the way our sources do, rather than in a consistent transcription system that might be better suited for sign languages (such as the Berkeley Transcription System in Slobin, 2006). While inconsistent coding inhibits comparison, one advantage of using the form presented in our sources is that sometimes this form is given in the ambient spoken language, and thus may relate articulatory information, since mouthing is common (Crasborn et al., 2008).

Sign languages can allow variety in order for the same range of reasons spoken languages do, including stylistic and grammatical concerns. So the murky issue of a so-called unmarked word order arises (Leeson and Saeed, 2012). We have chosen to be inclusive for fear of excluding relevant data. Still, we restrict ourselves to declaratives (as do most works in our survey and as do studies of spoken languages). A handful of our sources focus on interrogatives, so that few examples from them are of use to us. Importantly, even when a study is on some issue other than sign order, the data presented support our claims here (as, for example, with Inuit Sign Language, in Schuit et al. (2011), where they explicitly set aside order as an issue they will not address).

Further, we are leery of relying on data not taken from spontaneous conversation, given confounding influences of the laboratory situation itself. This concern is of particular weight for sign languages since Deaf linguistic consultants can be influenced by perceived researchers' expectations based on grammatical properties of the ambient spoken language (Deuchar, 1983, p. 76; Coerts, 1994). Nevertheless, we use data from all 42 languages regardless of how it was collected.

## Generalizations in the data

Here we list the generalizations we have found in the literature, augmented by our own observations of ASL and BSL conversations. These generalizations concern only MNPs, since all other nominals are expressed simultaneously to the V, precluding statements of linear ordering with respect to the V. So when we say S precedes V, we mean an S that is an MNP precedes the V, and so on. With the exception of the first, these generalizations are tendencies. The section A Comparison to Two Accounts discusses two accounts of these generalizations along with data that run counter to them.

### Generalization one

SOV is grammatical in all sign languages.

Yau (2008) makes this claim and our survey confirms it. We offer a typical example from Finnish Sign Language (Jantunen, 2008, p. 99):

BOY APPLE BUY ‘(The) boy buys an apple.’

If there are three MNPs in the sentence (which is uncommon in conversation) and all are arguments, then all can precede the V, as in this example from Israeli Sign Language (Meir et al., 2010b, p. 276):

WOMAN BOX TABLE PUT-ON ‘The woman puts the box on the table.’

### Generalization two

If an argument affects the phonological shape of the V, it precedes V.

This includes classifier predicates (Emmorey, 2003), agreeing verbs (Wilbur, 1987; Liddell, 1990), pointing verbs (De Langhe et al., 2004), spatial verbs (Padden, 1988; and see Liddell, 1990), and argument-sensitive verbs (Klima and Bellugi, 1979; Volterra et al., 1984, p. 33; a.k.a. “imitating” predicates, in Vermeerbergen et al., 2007b). (All types of V in this paper are discussed in Padden, 1988, 1990; Quadros and Quer, 2008; Padden et al., 2010.) Only plain Vs are exempt. Evidence comes from explicit statements by scholars in the surveyed articles and our own observations.

Many studies exhibit only SOV sentences and explicitly claim that V must come finally. Others exhibit only SOV sentences but claim that the order is topic-comment (as in McKee and Kennedy, 2005, on New Zealand Sign Language). Others explicitly claim that if the V is a classifier, it must come finally, while still others say a classifier predicate usually comes finally.

Many studies note sentences with the structure SVOV, the template of V sandwiches, where the two Vs indicate the same action. Whether we have two clauses here or only one is a tricky matter, but not one we need to resolve. What matters for us is that the first V is typically a simple form, whereas the second shows variable phonological shape, sometimes with aspectual marking on it, but often with more iconic information than the basic form, some of which may be affected by the arguments. (Many have noted for ASL that if a V is aspectually marked, its O precedes it even in single-V clauses, where the explanation involves raising the marked V to a right-branching functional projection, leaving the O in pre-verbal position, as in Chen Pichler, 2011; Fischer and Janis, 1990; Matsuoka, 1997; Braze, 2004.)

Here we see a V sandwich from Russian Sign Language where the second instance of the V is accompanied by a non-manual adverbial morpheme (Kimmelman, 2012, example 47):

                           face: doubtfully

LOOK G-R-U-Š-A          LOOK        ‘[He] looked at the pear

                                                        doubtfully’

Several studies explicitly mention that agreeing Vs come in final position. In Brazilian Sign Language, SVO is argued to be the unmarked order (Quadros, 2003) but agreeing verbs can also come in final position, with SOV order (see also Quadros and Lillo-Martin, 2010). If pointing verbs are discussed at all, they are typically mixed into the discussion of agreeing verbs.

We turn now to argument-sensitive verbs. The studies we consulted that offer evidence about argument-sensitive verbs (whether they note it or not) show that MNPs precede argument-sensitive Vs. For example, Johnston et al. (2007) discuss sentences containing HUG in Irish Sign Language, Flemish Sign Language, and Auslan. Sometimes the first appearance of an argument of HUG is an MNP which follows the V, as in this example from Auslan (Johnston et al., 2007, p. 192):

BOY MEET HUG_*p*_ GRANDMOTHER

We analyze the above as two clauses (as do the study authors), but significantly the first appearance of the O of HUG follows it (that is, GRANDMOTHER). And here the articulatory shape of HUG has not been adjusted to match the arguments. We indicate this fact with the subscript “p,” showing this is a plain V. (Argument-sensitive Vs, unlike most agreeing Vs, only optionally incorporate their arguments.) However, a V sandwich example from Irish Sign Language has two instances of HUG, the first without phonological adjustment for the arguments (HUG_*p*_) and the second with such adjustment (HUG_s_, where the subscript “s” indicates this is an argument-sensitive realization of the V). We find that the MNPs representing the relevant arguments (the hugger and the hugged) precede the second instance of HUG (HUG_s_) and, further, that the S precedes the O in this Irish Sign Language sentence (Johnston et al., 2007, p. 192):

BOY HUG_*p*_ WITH OLD-GRANDMOTHER HUG_*s*_

### Generalization three

The most common sentence type has only one new argument, which precedes the V.

We offer a typical example from Indian Sign Language (Aboh et al., 2005, p. 22) in the completive aspect (COMPL):

YESTERDAY FATHER DIE COMPL ‘Yesterday (my) father died.’

In fact, V S is generally unfound except when the V's sense introduces a player (which can be an event) onto the scene. Evidence for this generalization comes from explicit statements by scholars and our own observations. Additionally, we present evidence from so called split-sentence constructions.

#### Claims in the literature and our observations

First, sign languages usually express at most one MNP in a sentence, a fact some authors explicitly note. Many studies exhibit no V-initial sentences, again an observation often explicitly noted (and predicted in Minoura, 2008, p. 49, an idea proposed to her in personal correspondence by Susan Fischer). Other studies do have V-initial sentences, but the Vs function precisely to present or introduce a new argument, such as the existential verbs “seem,” “exist,” and the presentational verb “happen,” as in this example from Kenyan Sign Language (Jefwa, 2009, p. 167):

HAPPEN ONE MZUNGU COME KENYA ‘It happened one European came to Kenya.’

or possessives (some of which are presentational, see Kristoffersen, 2003; Johnston et al., 2007), as in this example from Swedish Sign Language (Bergman and Wallin, 1985, p. 219):

HAVE CAR I ‘I have a car.’

Still, in Malagasy Sign Language several V-initial assertions with other types of verbs are reported, an example being (Minoura, 2008, p. 52 and ff.):

MANDRARAKA KAMIÖ VATO ‘scatter truck rock’ ‘The truck scatters rocks.’

Minoura suggests the order in such examples is an influence from written Malagasy. (For remarks on the influence of written language order on sign order, see Fischer, 1975; Bogaerde and Mills, 1994; De Langhe et al., 2004; Milkovic et al., 2007; Yau, 2008; Wojda, 2010, who argues that this factor makes it impossible to determine the unmarked word order of Polish Sign Language.)

#### Split-sentence constructions

When one conveys a proposition in which the predicate has two arguments, and the referents of both are new to the conversation, a common tendency is to employ two clauses. The first introduces one MNP with a predicate that locates it or otherwise gives an identifying characteristic of it. That is, the first has a monadic V. The second clause introduces the other MNP with a dyadic V, that is, a V that takes two arguments. In the second clause the argument of the dyadic V that was introduced in the earlier clause is now not manually expressed.

In the first clause the MNP is the S of its clause per force. In the second clause, the MNP is typically the S. Very often, this second clause tells what the referent of the MNP in the second clause does to the referent of the MNP in the first clause. That is, the MNP in the first clause is coreferential with the O of the second clause (which is not manually expressed). This construction is known as “the split-sentence construction,” and has been characterized as S_1_V S_2_V, since each subject precedes its predicate, as exemplified here in Italian Sign Language (Volterra et al., 1984, p. 32):

BAMBINO SEDUTO MAMMA PETTINARE

child           seated       mother     comb

‘The child sits and the mother combs (his) hair.’

This signing stream conveys that the mother combs the hair of the seated child. The point for us is that instead of signing this proposition in a single clause with two MNPs, the choice is to have two clauses with only one MNP per clause, where that MNP is the S of the predicate and precedes it.

### Generalization four

When two MNPs occur in a locational expression that forms a single clause, larger more immobile objects tend to precede smaller more mobile ones, regardless of theta role or grammatical function.

However, animacy complicates the situation (see remarks in the section Order and the Visual Modality). We are appealing here to properties of the referents of the signs, not to properties of the signs themselves.

This fact is explicitly remarked on by many, and it is subsumed under the figure-ground principle (Happ and Verköper, 2006). An example from German Sign Language is seen here (the example is from Leuninger, 2000, p. 238; the translation is from Plaza-Pust, 2008, p. 85):

WAND_1_ JACKE ICH HANG_AN_1_ ‘I hang up the jacket on the

wall        jacket   I      hang-on                                        wall.’

### Generalization five

O is immediately adjacent to V.

Evidence for this comes from the order observed in the vast majority of examples in our survey. Certainly the order OSV occurs often in sign languages, but the literature overwhelmingly analyzes this as topicalization of O (indicated typically by prosodic cues and/or discourse contexts; Padden, 1988; Lillo-Martin, 1991; Petronio, 1993). This generalization supports the idea that there is a verb phrase (VP) in sign languages.

### Generalization six

In reversible sentences with plain verbs, SVO is favored.

Several studies note this tendency, regardless of the word order a language exhibits in non-reversible sentences. This tendency is noted so often that when a language does not exhibit it, the authors typically explicitly say that (as for Sign Language of the Netherlands, Coerts, 1994). Surprisingly, a study of Flemish Sign Language found more variation in word order in reversible sentences (where we find SOV and OSV) than in non-reversible (where we find only SOV) (Vermeerbergen, 1996). For the languages that favor SVO with plain verbs in reversible sentences, it would seem that NP_1_ V NP_2_ order is not ambiguous (interpreted only as SVO), whereas NP_1_ NP_2_ V order is open to the readings SOV and OSV (and see Fischer, 1975). In contrast, Kimmelman (2012) points out for Russian Sign Language, that, since OSV is marked, the cues that go with topicalization of the O should eliminate ambiguity in reversible sentences. The observation captured in generalization six remains, and we return to discussion of possible motivation in sections An Amodal Account and A Modal Account.

## A comparison to two accounts

We list the generalizations here for easy reference:

**Generalization One**. SOV is grammatical in all sign languages.
**Generalization Two**. If an argument affects the phonological shape of the V, it precedes V.
**Generalization Three**. The most common sentence type has only one new argument, which precedes the V.
**Generalization Four**. When two MNPs occur in a locational expression that forms a single clause, the larger more immobile objects tend to precede smaller more mobile ones, regardless of theta role or grammatical function.
**Generalization Five**. O is immediately adjacent to V.
**Generalization Six**. In reversible sentences with plain verbs, SVO is favored.

Taken together, we arrive at the generalization that SV is the order we find in most intransitive sign language sentences, and SOV and SVO are the orders for transitive sentences. Further, the choice between SOV and SVO is frequently determined by phonological considerations, where most of the time SOV should be preferred.

### An amodal account

One possible account of these generalizations is amodal: perhaps there are universal pressures on language that favor these patterns.

Consider generalization one. If we categorize languages by the six possible string permutations of S, O, and V, we find that together SOV and SVO characterize around 76% of spoken languages (Dryer, 2005), where SOV is dominant and SVO is not far behind. (For a current count, see Dryer's ongoing site http://wals.info/chapter/81. There, 41% of the 1377 spoken languages considered are SOV, and 35% are SVO.) Further, many V-initial languages also have an alternate word order with the S preceding the V, as in Arabic and Berber, in contrast to SOV languages, which tend to be strictly V-final in unmarked sentences (Tomlin, 1986; Herring and Paolillo, 1995; among many). We might therefore want to conclude that SOV or SVO is possible in all languages. The biggest problem for this conclusion is the Celtic family. Celtic languages have been claimed to be rigidly VSO except for main clauses in Breton and Cornish (Tallerman, 1998). There is not complete agreement on this, however. A drift toward SVO has been documented for Breton and Welsh (Raney, 1984; but see Willis, 1998 for Welsh), and a claim made that SVO is more frequent in modern Breton than VSO (Varin, 1979; but see Timm, 1989). We conclude that, on the whole, languages in general favor SOV, not just sign languages, and languages in general favor adjacency of V and O.

But the tendency for SOV is stronger in sign languages. Why? Some linguists argue that SOV is the default order for human language (including Givón, 1979; Newmeyer, 2000a). Newmeyer (2000b), in fact, claims SOV was the order in proto-language. Sign languages are young, so perhaps the acceptability of SOV in all sign languages follows. Indeed, could all the generalizations we noted in the immediately preceding section hold of young languages in general?

Many languages are known to have changed diachronically from SOV to SVO. In Indo-European, this is the case with English (Canale, 1978, among many), Greek (Taylor, 1994), Swedish (Delsing, 2000), Icelandic (Hróarsdóttir, 2000: p. 60), Norwegian (Sundquist, 2006), Spanish (Parodi, 1995), and Italian (Antinucci et al., 1979). (And see Fischer, 2010 for discussion of word order change in general, with a focus on Indo-European languages.) In Sino-Tibetan, this is the case with Bai, the Karen languages of Thailand and Burma, and may be responsible for a number of complex word order facts in languages of China (Dryer, 2003). In Niger-Congo, this is the case with Bantu languages (Givón, 1975). And the list continues. Rarely, however, do we find diachronic change in the opposite direction (Gell-Mann and Ruhlen, 2011). Some exceptions are the Austronesian language Motu (Crowley, 1992), the Western Oceanic language Takia (Ross, 2001), the Tai-Kadai language Kamti Tai (Khanittanan, 1986), and a few others, where that change is argued to be an influence from contact with an SOV language. (For overview and citations see Van Gelderen, 2011). Further, emerging sign languages favor SOV strongly (Meir et al., 2010b).

With respect to generalization three, while there is an enormous literature on (in)transitivity, trying to estimate the prevalence of different valencies is far from obvious (as in Brew and Schulte im Walde, 2002). In the substantial literature on creoles, no one, to our knowledge, discusses the relative prevalence of intransitive to transitive sentences (see, for example, McWhorter, 2000). And we are aware of no literature on any spoken language that claims that a particular language or language family has a tendency toward having only one fully referential NP (that is, an NP that is not a pronoun or an anaphor) in a clause or about young languages having such a tendency.

With respect to generalization four and spoken languages, again there is considerable literature on locational, existential, and possessive expressions, which have a number of semantic similarities. But much of that literature is concerned precisely with those semantic properties (for example, Hoekstra and Mulder, 1990). Some of the literature, however, addresses word order. Clark (1978, p. 88), for example, notes that “roughly speaking” definite NPs precede indefinite ones in English and French sentences of this type. However, we know of no claims to the effect that the size or mobility of the referent of an NP matters in the determination of word order in spoken languages.

One can also look to word order in spoken creoles with respect to the claim that young languages favor adjacency of V and O—that is, to support the claim that generalization five is true of young languages, since creoles are by and large young languages. DeGraff (2003, 2005) surveys a number of creoles and shows that, despite claims to the contrary (as in Bickerton, 1981, 1990 and following), creoles are not an exceptional kind of language morphologically and syntactically. In particular, SVO is not the (near) universal word order for creoles. Instead, creole VPs can be OV or VO. Still, it appears that many more creoles are SVO than SOV (Julien, 2002). So the evidence from creoles is not compelling with respect to the claim that young languages favor SOV (generalization one).

With respect to generalization six, while many languages allow a wide range of ambiguities, word order can be sensitive to situations of potential ambiguity with regard to grammatical functions (particularly S and O); indeed, sometimes in potential-ambiguity contexts in spoken language we do not find the otherwise expected word orders (Craig, 1977 for Jacaltec, Kuhn, 2001 for German, Lee, 2001 for Hindi and Korean, Vulanoviæ, 2005 and Flack Potts, 2007 for Japanese). Speakers of English adjust their word order to avoid ambiguity when the visual context is the source of the potential ambiguity (Haywood et al., 2005). While we have found no mention that this tendency is stronger in young languages, it certainly appears to be evidence of a natural language principle.

The only remaining generalization to be addressed with respect to spoken languages, number two, calls for a more complex discussion. The situation in spoken languages is interestingly complex, and we restrict the discussion here to the tense-carrying V (not to participles, which enter into a different paradigm). In general, for an argument to affect the phonological shape of the V (an effect that is arbitrary with respect to meaning for spoken languages—we return to this point in the section Order and the Visual Modality, when we discuss generalization two), there must be agreement between the two. Most commonly, if there is agreement, the V agrees with the S alone. Since S precedes V in most languages, this is no problem for our generalization. However, nearly 9% of spoken languages are V-initial (conflating the VSO and VOS examples on the site http://wals.info/chapter/81), among them the Celtic languages. In all the Celtic languages, the V does not agree with an S that is a fully referential NP, but it might agree with a pronominal S (whether overt or “pro”), as happens in Welsh (Borsley and Roberts, 2005, p. 40). But the very conditions for a pronominal S are that the referent already be present on the conversational scene. This is consistent with our motivation for generalization two. On the other hand, various varieties of Arabic allow both VSO and SVO order, but the V still agrees with the S even when the S follows the V, although interesting complications arise. In particular, in Standard Arabic (as opposed to Lebanese or Moroccan Arabic) when the S follows the V we find agreement for gender only, not for person and number, but when the S precedes the V, we find agreement for the full range of features (Aoun et al., 1994; Alexiadou and Anagnostopoulou, 1998).

Further, some languages allow agreement of a V with O, either direct object (as with Hungarian, Ge'ez, and Eastern Aramaic) or both direct and indirect object (as with Amharic, Swahili, and Lebanese Arabic), where O might well-follow V. Again, we find interesting complications. In Lebanese Arabic, where O follows the V, V can agree with an O only if it is definite (Koutsoudas, 1969). The same is true of Swahili (Givón, 1976). Since definite NPs are used when the referent is already on the conversational scene, generalization two seems to loom in the background again. On the other hand, in Amharic a definite O triggers agreement on the V, while an indefinite O does not (Baker, 2012), going exactly counter to our expectations if generalization two holds of spoken languages.

We have not done a survey of agreement facts in general, and agreement is remarkably messy (see Moravcsik, 1988). However, it seems clear that generalization two is not true of spoken languages, young or not, especially since we have found no typologists' claims to this effect.

In sum, an amodal account explains the preference for SOV, for the adjacency of V and O, and for word order to resolve potential ambiguities that arise in reversible sentences. But it does not account for the preference for clauses with only one fully referential NP, for word order in existentials and presentational sentences, nor for the phonological and semantic factors that affect word order in sign languages (i.e., generalizations two through four).

### A modal account

The alternative account we now consider is that these generalizations are a result of the modality of sign languages.

With respect to generalization one, a number of studies of gesture conclude that SOV is the default order in visual communication. In one study, Gershkoff-Stowe and Goldin-Meadow (2002) had English speakers describe scenes solely with gesture, and in another they presented speakers with pictures and asked them to order them in a way that would communicate a given scene. In both, people presented scenes in the order SMA—**s**tationary entity, moving entity, action. Importantly, the order of stationary before mobile entity is exactly what we find in sign languages, expressed in generalization four.

So et al. (2005) asked English speakers to describe vignettes in speech accompanied by gestures created on the spot as well as solely in gestures. When using gestures alone, the hands exploited space for reference and coreference more often than when speech was also used, and the types of entities the gestures represented differed. Most gestures accompanying speech concerned action, but gestures alone also concerned entities. From the data given, it appears that the order of “constituents” in gesture-only propositions resembles that in sign languages. For example, this is the description of a man communicating “man gives woman basket” with gestures (So et al., 2005, p. 1032):

He first set up one person (man) on his body [G1] and a second person (woman) on his right [G2]. He then produced a GIVE gesture moving from a location in front of him (later identified as basket) to the location to his right (woman) [G3, which was coreferential with G2]. After producing a gesture for basket in the location in front of him [G4, which was coreferential with G3], he again produced a GIVE gesture moving from the basket location to the woman location [G5, which was coreferential with G2, G3, and G4].

We see clearly the strategy of setting up participants in an action, then expressing the action. And, when relevant, we see the strategy of setting up the S before other participants. Importantly, we see that the action gesture, whose articulatory shape is affected by the participants, appears after those participants, just as in sign languages (see generalization two).

Goldin-Meadow et al. (2008) likewise find that SOV recurs in non-verbal communication. They had native speakers of languages with varying word orders (English, Turkish, Spanish, Chinese) perform studies like those in Gershkoff-Stowe and Goldin-Meadow (2002)—using wholly gestures in one study and arranging pictures in another, but now the scenes involved actions from an agent onto a patient (like transitive verbs) rather than intransitive changing-location actions. The order of constituents in speakers' native languages did not influence the order in these visual tasks. They conclude that SOV is the “natural order that we impose on events when describing and reconstructing them non-verbally and exploit when constructing language anew” (Goldin-Meadow et al., 2008, p. 9163).

Langus and Nespor (2010) replicated Goldin-Meadow et al.s' (2008) experiments with speakers of Italian and Turkish. Their results led them to a similar conclusion about the early stages of an emerging language: SOV is the preferred order in “simple improvised communication” (Langus and Nespor, 2010, p. 293). In another experiment they concluded that improvised communication does not organize its constituents hierarchically, in contrast to natural language. In a third experiment, they tested speech comprehension of sentences with prosodically flat words, where S, O, and V appeared in all possible orders and concluded that, while speakers understand best sentences whose order conforms to that of their native language (SVO for Italian; SOV for Turkish), compared reaction time in recognition of the meaning of speech strings with varying order shows a preference for V to precede O. They conclude that the computational system of grammar prefers SVO, whereas the preference for SOV in improvisational communication demonstrates “a direct link between the sensory-motor and the conceptual systems that prevails in gesture production” (Langus and Nespor, 2010, p. 308). In other words, SVO is the preferred syntactic order, with SOV being the natural conceptual order.

Gibson et al. (2013), in a gesture-production task with speakers of English, Japanese, and Korean, conclude that SOV is, indeed, the preferred order in gestural communication, but SVO arises when communication needs demand it, as in reversible events. The same is true in emerging sign languages; when asked to use gesture to describe reversible events in which both participants are animate (“girl kicks fireman”/“fireman kicks girl”), people prefer SVO (Meir et al., 2010a). This echoes the behavior of many sign languages, as stated in generalization six.

Gibson and colleagues tie this to works on language proper that claim SOV is the default order for human language. Their explanation for this shift to SVO in reversible events is based on the “noisy-channel” hypothesis (Shannon, 1948; Levy, 2008; Levy and Jaeger, 2007; the quote here is from Gibson et al., 2013, p. 1081).

A speaker wishes to convey a meaning m and chooses an utterance u to do so. This utterance is conveyed across a channel that may corrupt u in some way, resulting in a received utterance ũ. The noise may result from errors on the side of the producer, external noise, or errors on the side of the listener. The listener must use ũ to determine the intended meaning m. The best strategy for a speaker is thus to choose an utterance u that will maximize the listener's ability to recover the meaning given the noise process.

Languages need to be robust against this omnipresent noise. Essentially, a representation of an event with an animate patient is more robust to noise when the agent and patient are separated by the action (V). Spoken languages with SOV order can be robust against interfering noise by using case-marking, and they point out that case-marking is prevalent in SOV languages but almost absent in SVO languages.

Since languages are known to have changed diachronically from SOV to SVO, as discussed in section An Amodal Account, the idea that a noisy channel might be the impetus for such change arises. Hall et al. (2013) address this issue; they asked speakers of English to describe in pantomime both reversible and non-reversible transitive events. Critically, speakers always took on the role of actor, and Hall and colleagues noted what they call a “role conflict” in reversible events (Hall et al., 2013, p. 5):

To describe a non-reversible event (e.g., a woman lifting a box) using SOV order, participants would generally adopt the role of the agent (long hair), then produce a gesture for the box without adopting any role. In this case, the participant does not need to do anything special to re-inhabit the role of agent in time to produce the action gesture. In contrast, using SOV for reversible events (e.g., a man lifting a woman) is likely to entail a role conflict between O and V. For example, if a participant described a reversible event using SOV order, she or he would first adopt the role of the agent (flexing muscles), then the patient (long hair). The participant is now in the patient role but is ready to produce the action, which requires him or her to be in the agent role. If the participant were to produce an action gesture without first doing something to switch back into the agent role, it may “feel” to him or her as if it is the patient and not the agent that is carrying out the action. It is this that we refer to as role conflict.

They suggest that the preference for SVO in reversible events is due to a desire to avoid role conflict. And they note that when speakers do produce SOV order in reversible events, they find ways to get around the potential role conflict, either by not embodying the role of the patient (perhaps simply tracing it in space) or by establishing a spatial location for agent and another for patient and then shifting appropriately between them when they pantomime the action. (Spatial marking is also observed in Gibson et al., 2013, who compare it to case marking in spoken languages.)

Schouwstra (2012) also addresses the issue of a natural word order by looking at gesture in an improvised communication experiment. Many of her findings echo those of earlier scholars. Her work differs, however, in arguing that constituent ordering is influenced not only by the cognitive abilities involved in making an analogy between language meaning and cognitive representations (and see de Swart, 2009), but also by the communicative needs involved in public expression, where the conventional nature of language imposes itself (Roberge, 2009). Participants view an event on a screen. Then they use gesture to describe it. The process of transitioning from the simultaneity of the picture to the linearization of the gesture string forces participants to consciously choose the order in which they present things. This choice can be made on grounds of communicative needs. Schouwstra makes a distinction between “motion events,” which involve extensional predicates (that create transparent contexts), such as *carries* in “princess carries vase,” and “intensional events,” which involve intensional predicates (that create opaque contexts), such as *think of* in “cook thinks of sock”. Both Turkish and Dutch speakers strongly preferred SOV order in their gestural representation of motion events, but SVO order (though less strongly) in their gestural representation of intensional events. Schouwstra then looked at order in events involving a subset of intensional predicates, the creation verbs. She found that the tendency toward SVO was smaller with creation verbs than with other intensional verbs but was still the preferred order. (Indeed, we found evidence of pressure toward SVO with creation verbs in our study, but nothing conclusive.) There is no doubt that semantics influences word order in these experiments. As Schouwstra (2012, p. 148) says, “When making a sequence of the different elements, they [the participants] are forced to impose an order on the information. So it is only in making the information public, in being involved in communication, that ordering plays a role.” Likewise, she found that when people interpret gesturing of others, SOV strings are interpreted more often as motion events than SVO strings are, and SVO strings are interpreted more often as intensional events than are SOV strings. “This shows that in emerging communication systems, meaning and structure have more to do with each other than previously thought. Moreover, it suggests that ordering information in utterances in these systems is quite an active process, rather than simply a reproduction of how information is represented mentally” (Schouwstra, 2012, p. 148).

Christensen and Tylén (2013) offer another gestural communication experiment which uses an interactional paradigm instead of an elicitation task. Participants communicate to a passive experimenter or a camcorder, thus participating in proper bidirectional communication, where dyads are dependent on mutual comprehension of the gestural systems that evolve during the experiment sessions. They followed up on Schouwstra's work, contrasting “object manipulation events” to “construction events,” the latter of which involve effective verbs. The former consistently yielded SOV order, while the latter yielded SVO order, as we also found for sign languages, but with far too few examples to base a generalization upon. Again, we see that event structure rather than a cognitively natural order influences order in these gestural strings.

So the data on gestural communication is consistent with all the generalizations of section Generalizations in the Data.

Further, homesigners often produce strings of V plus one argument, where they place the V finally (that is, SV or OV) (Goldin-Meadow, 2003). And studies of young sign languages, still with a relatively unstable grammar, reveal a tendency for utterances to consist of SV, OV, and SOV (Senghas et al., 1997; Sandler et al., 2005; Haviland, 2011). These findings are, so far as they go, consistent with the generalizations of section Generalizations in the Data.

### Conclusion

The amodal account covers some of our generalizations; the modal account covers all. One might then conclude that our observations on sign languages are evidence of a natural visual order. That is, we know vision is at play in both producing and receiving gestural strings and sign languages, so if one is to claim some other cognitive ability is at play, the burden of proof lies on them.

Nevertheless, the fact that visual communication (gesture and sign languages) and spoken languages, particularly young languages, share important tendencies in order of constituents should make us wary of such a conclusion. It seems unlikely that totally independent pressures on sign languages and spoken languages would happen to produce such similarities. Two logical possibilities come up. One is that the pressures evidenced in the generalizations about order in sign languages really do hold of language in general, but that over time evidence for several of them has been lost as these pressures yield to competing pressures (whatever they might be), or several of them are simply gapped in spoken language. This possibility is not open to testing, unfortunately, but the speculation remains (and see Hale, 1975 for discussion of gaps in universals).

The other possibility is that the word order generalizations for sign languages reveal universal pressures augmented by visual pressures. As Chomsky (2013, p. 35) says, “… each language incorporates a mechanism that determines an infinite array of hierarchically structured expressions that are transferred for interpretation to two interfaces: the sensorymotor system SM for externalization and the conceptual-intentional system CI, for thought (broadly understood).” The structured expressions in spoken and sign languages are transferred to different sensorimotor systems—leading to different realizations.

At this point one might be led to the reasonable position that the universal pressures on word order discussed in this paper are grammatical in nature, while the pressures that apply only to sign language word order are visual in nature. Still, there is a way to see a coherence in the two sets of pressures. If, in fact, pressures of both the auditory and visual systems are behind the universal pressures on word order, we can view the sensorimotor pressures as motivating this particular part of universal grammar, which is apparent in both spoken and sign languages. Certainly, biological sources as a foundation for universal grammar should be seriously examined. After all, the innate language faculty, which serves for both spoken and sign languages, evolved somehow.

Given that language is embedded in the neuronal circuitry of the brain, and given that motor, cognitive, and perceptual systems are implicated in language learning and language use, we may assume that the language faculty should have come from pre-existing competencies, which initially were unrelated to language (Cowie, 2008; and, for compatible remarks, see even non-nativists, such as Tomasello, 2003). Certainly, finding evidence today that bears on human cognitive evolution is a daunting job, but our findings here suggest that comparative studies of languages in different modalities may offer new ways to approach the issue (and see Napoli and Sutton-Spence, 2011). Whatever the truth about language evolution may turn out to be, the birth of the language faculty will have been complex and, if we are correct, will involve many other competencies that developed earlier and were then adapted to language, with the sensorimotor systems playing a significant role.

The idea that shared language properties may follow from shared pressures of the visual and auditory sensorimotor systems seems to be gaining strength in the neuroscience field. Tettamanti and colleagues argue (2005, p. 273), “… listening to sentences that describe actions engages the visuomotor circuits which subserve action execution and observation” (but see Mortan Ann Gernsbacher's remarks in Gallese et al., 2011). Further, the prevalence of SOV and SVO accords well with the representation of action in Broca's area (Kemmerer, 2012; but for arguments that Broca's area does not have a unified function, see Fedorenko et al., 2012). Additionally, neural tissue involved in language processing involves polymodal neural activity, so the idea that the different sensorimotor systems would share properties may follow from a cooperation of these neural activities (Petitto et al., 2000). And, finally, there is evidence that intellectual and perceptual-motor skills involve hierarchical unpacking of chunks of knowledge (Rosenbaum et al., 2001; Rosenbaum, 2009; Clark, 2013), thus sensorimotor-system pressures may even motivate the hierarchical nature of universal grammar.

Further, if this sensorimotor hypothesis about word order can be supported, it is the more interesting one since it calls for a reassessment of how to approach the issues of the order of the major constituents in language in general. Let us assume that the grammar of all languages organizes words into phrases, including VP. That means that OV and VO are both generated, depending on whether phrases in the language are head-initial or head-final. So the potential orders we can expect the relevant sensorimotor systems to produce in both spoken and sign languages for transitive sentences are SOV, OVS, SVO, and VOS. The fact that SOV and SVO occur so frequently in spoken language and so overwhelmingly in sign languages suggests that pressures of the sensorimotor systems favor S preceding VP. This accounts for the infrequency of spoken languages with unmarked word order being OVS (under 0.8%; 11 out of 1377) and VOS (under 2%; 25 out of 1377); they are bucking the sensorimotor system pressures. This also leads to the conclusion that OSV will be the result of topicalization from either SOV or SVO. That is, OSV should be a marked order in language, calling for contexts in which we are somehow highlighting the O. In fact, only 4 spoken languages out of 1377 have been claimed to have OSV as unmarked order (under 0.3%).

Finally, let's consider VSO. An immediate problem is that V and O are not adjacent. Further, we see no evidence of pressure from sensorimotor systems to favor V in initial position. As we discussed, VSO is (almost) non-existent in sign languages and is rare as an unmarked order in spoken languages (under 7%, 95 out of 1377). Importantly, as also discussed, VSO in spoken languages often has SVO as an alternate order. The strong consensus in the literature is that VSO arises from SVO via V-raising in order to satisfy requirements of the grammar (Choe, 1987; Carnie and Guilfoyle, 2000; for example), even for Irish (Bobaljik and Carnie, 1992). (For details on the analysis, see Alexiadou and Anagnostopoulou, 1998).

The sensorimotor hypothesis, then, says that S precedes VP as a fundamental strategy in language. This conclusion finds support in the language of people who are linguistically deprived in the sense that they were not exposed to accessible language during the early years of life. Such people generally manage to use appropriate word order in most situations, whereas many other properties of language are problematic for them. This is true of Genie, an abused girl who was not rescued until the age of around thirteen (Curtiss et al., 1973; Fromkin et al., 1974; Curtiss, 1977; Goldin-Meadow, 1978) and of deaf “late learners” (Johnson and Newport, 1989; Newport, 1990; Newport et al., 2001; Wood, 2010). In fact, deaf children first exposed to ASL after the age of 6 do not produce appropriate variations in word order that native signers produce (even as young as 2 year olds), instead using SVO heavily (Lillo-Martin and Berk, 2003). That it is SVO rather than SOV that these late learners produce is consistent with the fact that their morphology is underdeveloped, thus their verbs exhibit fewer instances of phonological shape affected by arguments (that is, fewer instances of the situations that call for SOV, see discussion in section Generalization Two) than verbs of native signers (Newport, 1991). Thus, the sensorimotor hypothesis accounts for why some characteristics of language are “resilient” and others are “fragile” (Wood, 2007, 2010); the resilient ones are dependent upon sensorimotor pressures that exist regardless of language and that motivate certain parts of the grammar, while the fragile ones are not. In other words, late learners look at the world visually and their language is sensitive to visual pressures. On the other hand, they have trouble producing those grammatical structures that are not motivated by sensorimotor pressures, but are arbitrary to the particular language.

Given this explanatory force of the sensorimotor hypothesis, it is worth taking a closer look at what some of these pressures might be. The sensorimotor account of word order amounts to saying there are universal pressures driving the order similarities among sign and spoken languages, pressures that are imposed by factors that the visual and auditory sensorimotor systems have in common, and there are modality-specific pressures resulting in the order differences between sign and spoken languages, pressures imposed by the visual (-manual) sensorimotor system. In the next section we explore the relevant visual pressures on sign languages, and one suggestion of a pressure imposed by the manual articulators.

## Order and the visual modality

Here we consider the generalizations that hold of sign languages but not of (young) spoken languages (i.e., generalizations two through five), and we argue each follows from visual needs or principles. Some of our accounts rely on coherence and iconicity; they turn upon the construction of a visual image, making testable predictions. There is no doubt that iconicity plays a role in sign language order. As De Langhe et al. (2004, p. 117) say (in our own translation), “… the most important thing for constructing sign expressions is iconicity… one must find the image that represents the subject and as soon as an image is formed in the mind, the translation into sign language becomes clear and easier.” Thus, there is pressure for temporal and spatial organization to work together coherently at every level of grammar, maximizing comprehensibility.

### Generalization two

If an argument affects the phonological shape of the V, it precedes V.

Why should sign languages but not spoken languages share this generalization? In a spoken language, the relationship between phonological features and meaning is (to a huge extent) arbitrary. In a sign language, that relationship is not arbitrary. Instead, the phonological shape of classifier predicates, agreeing verbs, pointing verbs, and argument-sensitive verbs will vary in non-arbitrary ways according to meaningful properties of their arguments, such as their spatial index and their size, shape, and general category (human, animal, small round object, and so on). For example, agreeing Vs involve a transfer of something (abstract or concrete) from one location to another. If visual perceptibility matters to the order of arguments, then we might expect an alignment such that the visual representation of transfer should involve a path that moves in the direction of the transfer. That is, the spatial-temporal organization should be coherent with the semantics of the utterance. This means that the point of initiation of the movement should be spatially indexed with the argument that is the origin of the transfer, and the endpoint of the movement should be spatially indexed with the argument that is the goal of the transfer (Meir, 1995; Aronoff et al., 2003). In most of the sign languages we have read about with respect to Vs of giving and taking, the verb GIVE moves from a point indexed with the giver to a point indexed with the receiver of the gift; whereas the verb TAKE moves from a point indexed with the one (or the place) from whom something is taken to a point indexed with the taker. In such examples as in classifier constructions, we find “mappings of envisioned mental spaces onto signing space” (Taub, 2001, p. 163). If the addressee is to make sense out of the phonological shape of these predicates, including the direction of path movement, the relevant arguments should already be present in the discourse or be introduced within the sentence before the V (for a similar claim, but with more conditions on it, see Yau, 2008, pp. 152–153).

With respect to classifier constructions, the non-arbitrariness of phonological features is rampant. To express that someone almost gave something to someone else, one might move only halfway along the path from one spatial index to another, for example (Quadros and Quer, 2008), perhaps with a dynamics that portrays hesitancy. Thus, iconicity can be a motivation with respect to the order of elements and with respect to various factors of a predicate's movement (direction, length of path, and so on), as well as with respect to other phonological parameters (such as facing of the hands, as in Meir, 2002). Syntactic structure is here a linguistic construction that itself conveys meaning (Goldberg, 1995, 2003).

As final evidence that generalization two reflects semantic concerns that are realized visually, we note that sign languages, like spoken languages, can exhibit phonological feature-spreading rules (as in compounding in ASL, see Liddell and Johnson, 1986). Such rules are purely phonological; they are arbitrary with respect to semantics, and in these instances feature spreading can be anticipatory as well as perseverative. So when phonological shape is arbitrary, signs can be affected by what follows linearly. It is only when phonological shape is meaningful (as with classifier predicates, agreeing verbs, pointing verbs, and argument-sensitive verbs) that the element that influences the phonological shape appears beforehand as the unmarked order.

Certainly it is possible to articulate a predicate whose phonological shape is affected by an argument before introducing the relevant argument (Padden, 1988), but this order is marked. The effect, according to the linguistic consultants we have asked, is like holding back information for dramatic impact and then revealing it, as in *And in walked…. her husband!*

An example from Inuit Sign Language makes the point nicely (Schuit et al., 2011, p. 21):

INDEX-LOC_3*a*_ SCOOP DRILL-HOLE-WITH-AUGER FINISH.

_3*a*_WALK_1_      TAKE-LONG-ITEM     _1_WALK_3*a*_      WHITE-MAN

CHISEL_V_.    DROP     LONG-THIN-OBJECT   MOVES-BELOW-

SURFACE.

‘Over there they started a hole with a scoop, and then drilled it with an ice-auger. Someone walked from there towards me and took my chisel. The white man walked back (to the hole) and used the chisel. Then he dropped it, and it went all the way to the bottom (of the sea).’

(The translation is Schuit et al.'s, but the following comments are ours.) In the second sentence, “_3a_WALK_1_” indicates that someone walked from spatial location 3a (where the scoop and then the ice-auger were used) to spatial location 1 (which is the signer's location). “TAKE-LONG-ITEM” indicates a classifier predicate in which someone is taking hold of a long item. “_1_WALK_3a_” indicates that someone walked from spatial location 1 back to spatial location 3a. And only now are we told that the someone was a white man and that the long object he took was the signer's chisel. Here an unspecified NP is spatially indexed; we can't see who it is, all we see is that he picked up something long. Then we see it's a white man and we realize what he picked up is, in fact, a (the signer's) chisel (from how he used it). The word order reflects clarification after the fact. That is, the spatial index (3a) and the classifier predicate (TAKE-LONG-ITEM) precede the information about who was in that spatial index and what long item that classifier predicate involves. The MNPs come late for dramatic impact.

Russian Sign Language presents a (partial) exception to generalization two. SOV is found with classifier predicates, whereas SVO is found with plain verbs, as we expect. But SVO is also found with agreeing verbs (Kimmelman, 2012). And Volterra et al. (1984) report for Italian Sign Language that in non-reversible sentences, SOV is used only if the V is a classifier or somehow else incorporates the O. However, they also say that SVO, the unmarked order, can occur under the same conditions (but see Cecchetto et al., 2006 for the analysis of Italian Sign Language as SOV).

### Generalization three

The most common sentence type has only one new argument, which precedes the V.

The fact that the lone argument tends to precede the V is shared by (young) spoken languages. What's not shared is a particular strategy that sign languages often employ. Essentially, sign languages tend to put the relevant players on the stage one at a time, focusing our attention with a single spotlight on a single player, then moving that spotlight to a second player, and so forth. We saw that same strategy in gestural strings and in homesign (discussed in section A Modal Account).

Possibly, this is a visual strategy. While the retina can receive much information (our visual environment is typically cluttered), at a given time, only a small amount of that information can be processed. “Subjectively, giving attention to any one target leaves less available for others” (Desimone and Duncan, 1995, p. 193). By introducing only one argument per predicate, we increase the chance that each argument will get attention, enhancing good communication of the event. Nevertheless, signers can convey information simultaneously with multiple articulators (both hands, various parts of the face). So we are not convinced this is a visual strategy.

More likely, it is a manual strategy. The manual articulators move slowly in comparison to the speech articulators, which means it takes time to set things up. So once we have the stage set, there's no need to keep doing something as uneconomical as repeating information everyone already knows.

### Generalization four

When two MNPs occur in a locational expression that forms a single clause, the larger more immobile objects tend to precede smaller more mobile ones, regardless of theta role or grammatical function.

Among others, Volterra et al. (1984, pp. 35, 38) suggest this ordering is a direct result of the visual modality because larger objects are perceptually more important, a suggestion supported by a study on the order of gestures (not signs) in which participants consistently place a gesture for a larger stationary object before a gesture for a smaller moving one (Gershkoff-Stowe and Goldin-Meadow, 2002). On the other hand, in both existential and locational sentences animate objects tend to precede inanimate ones (although see Coerts, 1994 and Kristoffersen, 2003 for complications), and sometimes these two principles conflict, which is the explanation these studies give for freer word order in existential/locational sentences, and which is the reason why we did not offer a separate generalization about word order in existential/locational sentences in particular.

A sign utterance that conveys relative spatial information about two objects creates that information spatially and, thus, evokes a cognitive representation of those objects in those spatial positions. It appears that, with respect to that evoked representation, sign languages are sensitive to the relevant visual principles. Studies show that perception of small objects (under 10 cm) differs from perception of large objects (Pakhomova, 2000). Further we perceive small objects as moving more quickly than large objects even when they are moving at the same rate (Leibowitz, 1965). So the fact that existential/ locational sentences tend to establish the location of large objects before they establish the location of small objects appears to follow from some property of visual perception.

### Extra comment on generalization five

O is immediately adjacent to V.

Since this generalization holds of most spoken languages (which we expect, given the existence of VP) and of creoles (i.e., young languages) as well as sign languages, pressures common to all sensorimotor systems apply here. But Meir (2002) points out that in Israeli Sign Language a V can agree with its O without agreeing with the S, a situation not found in spoken languages. This suggests that the visual modality adds pressure for a visual unity or coherence of the V and O in sign languages.

### Conclusions about visual and manual pressures

Sign languages are subject to the universal pressures on all languages. Some of those pressures are common to auditory and visual sensorimotor systems and, thus, we suggest they motivate parts of universal grammar. But sign languages are also subject to visual and perhaps manual pressures that set them apart from spoken languages. That sign languages should fall together typologically with respect to various aspects of grammar is not a new claim. For example, all sign languages use simultaneous expressions, a fact most often accounted for by the slowness of the manual articulators (Hohenberger, 2007). By recognizing visual pressure on sign order, we can see that sign languages exploit simultaneity not simply because they can (given that spoken languages can, too—Napoli and Sutton-Spence, 2010), nor totally because of the timing needs due to slow articulators, but because by exploiting it they can better align syntax and semantics with a visual coherence that is at the core of signing itself.

Our study argues that all sign languages will organize signs at the sentence level in a similar way partly because that's how all languages would do it, all else being equal, and partly because the visual modality entails creating pictures. Certainly these pictures are iconic in only the most abstract of ways and that iconicity is concentrated in the productive much more than in the frozen part of the lexicon (Klima and Bellugi, 1979; McDonald, 1985; Brennan, 1990; Taub, 2001; Russo, 2005; Cuxac and Sallandre, 2007; Sallandre, 2007; Konrad, 2011), otherwise any sighted person would be able to understand any sign language. Indeed, in the frozen lexicon, many signs are opaque in that their meanings are not guessable at all. And with respect to the others, signs whose meanings “are most directly interpreted from visibly present referents” or “can be shown by pantomimic expression” are more likely to be understood relatively accurately by people who do not know the given sign language than are signs whose meanings involve some kind of “metonymic association” or are “more culturally specific” (Boyes Braem et al., 2002, p. 187).

But once particular frozen lexical items are understood, and once one understands the nature of all the various types of predicates in sign languages, the organization of frozen and productive signs in the visual space and time of a sign sentence can be seen as largely iconic, where recognizing this iconicity calls for analogy, metaphor, metonymy, and other complex cognitive activities (Napoli and Sutton-Spence, 2011). So the signed creation of pictures demands a visual coherency in order to be interpretable, and this demand for visual coherency should be equally high in any sign language.

## Manual factors

A few of the studies we cite claim that manual considerations are relevant to word order. Nadeau and Desouvrey (1994, p. 156), in their study of Quebec Sign Language, suggest that SVO is favored for “mechanical” reasons, claiming that any other order would require additional transitional movements between the signs. Fischer (1975) mentions manual reasons for expecting the SOV order of ASL to change to OSV over time. Two studies point out that the O referred to in a handling classifier must immediately precede the classifier predicate (Jantunen, 2008 for Finnish Sign Language, Sze, 2003 for Hong Kong Sign Language in non-reversible sentences). We leave these remarks for future investigation.

## Implications

Universal pressures and visual pressures conspire to bring about the generalizations we have found. We promoted the position here that those universal pressures follow from shared characteristics of the auditory and visual sensorimotor systems and we suggested that those shared characteristics are part of the motivation for universal grammar. Further, as visual pressures, in particular, play a stronger role in sign languages than in spoken languages, they mediate the emergence of the grammars of sign languages in such a way that sign languages tend to converge on a shared design that is, in the respects discussed in section Order and the Visual Modality, different from spoken languages.

We conclude that SOV and SVO should be the prevalent orders found in all declarative sentences in sign languages and that V-initial sentences should be restricted to presentational or existence sentences. In all of this, recall that we are talking only about the distribution of MNP arguments with respect to V. In fact, plain verbs are the only type that should show variation among languages in unmarked word order, specifically between SOV and SVO. That's because plain verbs are the only verbs whose phonological shape is not affected in an iconic way by their arguments. And, as it turns out, SOV and/or SVO are the unmarked orders for plain verbs across all the languages in the studies we examined (see remarks at the end of section Generalization Four under Order and the Visual Modality).

The account of sign order in sign languages that arises from our survey of the data in many studies needs to be tested through examination of a large video corpus, something that has not been possible for most linguists thus far. Fortunately, three major data corpora have recently been made available, for British Sign Language (BSL corpus project, discussed in Schembri, 2008), Auslan (Johnston and Schembri, 2007b; Johnston, 2008, 2010), and Sign Language of the Netherlands (Crasborn and Zwitserlood, 2008). Similar databases are under construction, including for German Sign Language (Hanke et al., 2010), Italian Sign Language (Branchini et al., 2009), Chinese Sign Language (Zhang et al., 2013), and French Belgian Sign Language (Meurant and Sinte, 2013). These databases can serve as a model for building databases for other sign languages, and they will enable researchers to make headway on linguistic analysis with confidence in the foundation upon which arguments are constructed and to both pose and answer questions regrettably infeasible without such a base. We offer our remarks here then, as a starting point for examining sign order with the goal of understanding better the sensorimotor system pressures affecting that order.

## Author contributions

All parts of this work were done through collaboration of both authors.

### Conflict of interest statement

The authors declare that the research was conducted in the absence of any commercial or financial relationships that could be construed as a potential conflict of interest.
